# Cost-Minimization Analysis of Tolvaptan Treatment for ADPKD in Southern Spain

**DOI:** 10.1016/j.ekir.2024.10.024

**Published:** 2024-10-26

**Authors:** Francisco J. Roca Oporto, Jaime Espín, Cristina Andrades Gómez, Gema Montilla Cosano, Maria Cristina Sánchez-Pozo, José L. Rocha

**Affiliations:** 1 Unidad de Gestión Clínica Nefrología, Hospital Universitario Virgen del Rocío, Sevilla, Spain; 2 Escuela Andaluza de Salud Pública, Granada, Spain; 3 Instituto de Investigación Biosanitaria, Granada, Spain; 4 CIBER en Epidemiología y Salud Pública (CIBERESP), Spain/CIBER of Epidemiology and Public Health (CIBERESP), Madrid, Spain; 5 Unidad de Gestión Clínica Laboratorios Clínico, Hospital Universitario Virgen del Rocío, Sevilla, Spain; 6 Universidad de Sevilla, Departamento Medicina, Nefrología, Sevilla, Spain

To the Editor:

Autosomal dominant polycystic kidney disease (ADPKD) is a chronic and progressive disease that places an economic burden on the health care system and patients.^1^ The cost of kidney disease increases with the degree of kidney failure.^2^ Slowing the progression to end-stage renal disease is the main goal of autosomal dominant polycystic kidney disease treatment. During our 3-year prospective study conducted at the Virgen del Rocío University Hospital,^3^ we adjusted the tolvaptan dose according to the 24-hour urinary osmolality. A cost-minimization analysis was performed (Supplementary Methods).

### Results

Forty patients were included in this study for 3 years. All participants received an initial tolvaptan dose of 45/15 mg, and this dose was increased to 60/30 mg for 5 patients. Using the initial price of tolvaptan, the annual cost of treatment with a dose of 45/15 mg was €37,437, and that with a dose of 60/30 mg was €40,432 (Supplementary Table S1). In Table 1, we show the annual costs incurred with the maximum dose of tolvaptan (90/30 mg), with the average dose of the TEMPO 3:4 trial (reference study), and with the average dose of the TEMPO 3:4 trial with dropouts (dropout rate of 22%).^4^ A comparison of the incremental costs of treatment comprising the urinary osmolality–based dose with those of the maximum dose of 90/30 mg showed that the cost differences during 3 years of treatment would have been €623,003 if the dropout rate of our study was considered, €460,657 if the average dose of TEMPO 3:4 had been considered, and €124,402 if the TEMPO 3:4 dropout rate was considered (Table 1).

**Table 1:** Treatment cost during the study and incremental cost compared with the cost of tolvaptan therapy until 2021

Different cost situations	Tolvaptan price until 2021				Savings over the course of 3 years with OsmU-based therapy
	Baseline *n* (40)	Year 1 *n* (38)	Year 2 *n* (38)	Year 3 *n* (34)	
Tolvaptan dose (mg), median (SD)	60 (40)	60 (35)	60 (33)	60 (30)	
	90 (0)	90 (3)	90 (5)	90 (4)	
Cost patient/year with OsmU-based therapy, Euros	0€	1,431,614€	1,437,604€	1,284,859€	
Annual budgetary impact, euros	0€	+1.431,614€	+5990€	-153,745€	
Theoretical patient-year cost with 90/30 mg, Euros	0€	1,650,264€	1,650,264€	1,476,552€	
Incremental cost compared with that of OsmU-based therapy, Euros	0€	+218,650€	+212,660€	+191,693€	+623,003€
Annual cohort cost using TEMPO 3:4 data, Euros	0€	1,594,181€	1,594,181€	1,426,372€	
Incremental cost compared with that of OsmU-based therapy, Euros	0€	+162,567€	+156,577€	+141,513€	+460,657€
Annual cohort cost with the TEMPO 3:4 dropout rate, Euros	0€	1,550,151€ (*n* = 37)	1,427,172€ (*n* = 34)	1,301,156€ (*n* = 31)	
Incremental cost compared with that of OsmU-based therapy, Euros	0€	+118,537€	-10,432€	+16,297€	+124,402€

OsmU, urinary osmolality.

### Discussion

The cost of tolvaptan after adjusting the dose according to urinary osmolality allowed annual savings of €100,000 to €200,000 for our cohort of 40 patients compared with the cost of using the maximum tolerated dose of tolvaptan (90/30 mg) or the average dose used during the TEMPO 3:4 trial. A comparison of the costs of treatment and chronic kidney disease, especially renal replacement therapy, indicated that the prevention of autosomal dominant polycystic kidney disease progression would result in economic savings. Our results suggest that significant economic savings can be achieved when the dose of tolvaptan for autosomal dominant polycystic kidney disease is adjusted according to urinary osmolality.

## Disclosure

All the authors declared no competing interests.

## Data Availability Statement

The data that support the findings of this study are available from the corresponding author upon reasonable request.

## Supplementary Material

Supplementary File (PDF) **Supplementary Methods. Table S1.** Costs of the original tolvaptan and generic tolvaptan.

## Footnotes

Supplementary File (PDF)

**Supplementary Methods.**
**Table S1.** Costs of the original tolvaptan and generic tolvaptan.

## References

[1] Lentine K.L., Xiao H., Machnicki G., Gheorghian A., Schnitzler M.A. (2010). Renal function and healthcare costs in patients with polycystic kidney disease. Clin J Am Soc Nephrol.

[2] Jha V., Al-Ghamdi S.M.G., Li G. (2023). Global economic burden associated with chronic kidney disease: A pragmatic review of medical costs for the inside CKD research programme. Adv Ther.

[3] Roca Oporto F.J., Andrades Gómez C., Montilla Cosano G., Aguilera A.L., Rocha J.L. (2024). Prospective study on individualized dose adjustment of tolvaptan based on urinary osmolality in patients with ADPKD. Kidney Int Rep.

[4] Torres V.E., Chapman A.B., Devuyst O. (2012). Tolvaptan in patients with autosomal dominant polycystic kidney disease. N Engl J Med.

