# Supplementary material for: Cost-Minimization Analysis of Tolvaptan Treatment for ADPKD in Southern Spain
Source: Kidney Int Rep. 2024 Oct 26;10(1):265–6. doi: 10.1016/j.ekir.2024.10.024 (PMC11725810; doi:10.1016/j.ekir.2024.10.024)
Supplement: Supplementary File (PDF) — Supplementary Methods. Table S1. Costs of the original tolvaptan and generic tolvaptan. [file mmc1.pdf]

Supplementary Material

Supplemental Methods.

Supplemental Table S1. Costs of the original tolvaptan and generic tolvaptan.

Supplemental Methods

Analysis

This cost-minimisation analysis was performed to quantify the savings that could be derived from the use of a lower dose of tolvaptan without the loss of efficacy. We investigated the economic impact of the use of tolvaptan doses adjusted based on OsmU. Three doses of tolvaptan with a divided regimen were administered to patients with ADPKD (45/15 mg, 60/90 mg, and 90/30 mg); one dose was administered in the morning and one dose was administered in the afternoon. The initial tolvaptan dose was 45/15 mg; however, it was increased if the OsmU was >200 mOsm/kg for 2 consecutive months. Based on the results of our study, an analysis of two doses, 45/15 mg and 60/30 mg, was performed. These results were compared with those obtained using the doses and dropout rates of the TEMPO 3:4 trial after adjusting for our study population.

Costs

The official prices of tolvaptan (retail prices plus value-added tax) were obtained using Bot Plus online software and a database of health information regarding medicines in Spain prepared by the General Council of Official Colleges of Pharmacists. All costs were assessed as 2021 euros (€) because our final prospective study was completed at that time. In Spain, tolvaptan is 100% financed by the National Health System; until June 2021, a generic version of tolvaptan was not available. Although our study was conducted between 1 January 2017 and 31 December 2021.

Population

The patient population comprised those with ADPKD with rapid progression treated at the Virgen del Rocío University Hospital in Seville, Spain. The criteria for inclusion were age older than 18 years, CKD caused by ADPKD stages G2 and G3, and rapid

progression. All patients initiated tolvaptan treatment between 1 January 2017 and 31 December 2021. All study participants provided written informed consent according to a research protocol approved by the Hospital's Research Ethics Committee in accordance with the requirements expressed by the principles of the Declaration of Helsinki and the principles of the Declaration of Istanbul.

Table S1. Costs of the original tolvaptan and generic tolvaptan

	Tolvaptan (price until 2021)			Tolvaptan (price after the introduction of generic version)		
	**60 mg**	**90 mg**	**120 mg**	**60 mg**	**90 mg**	**120 mg**
**Average unit price (PVL+VAT)**	51·99€	56·10€	60·30€	25·99€	28·07€	30·15€
**Dose**	2 tablets/day (45/15 mg)	2 tablets/day (60/30 mg)	2 tablets/day (90/30 mg)	2 tablets/day (45/15 mg)	2 tablets/day (60/30 mg)	2 tablets/day (90/30 mg)
**Cost per day**, euros	103·99€	112·31€	120·63€	51·99€	56·10€	60·31€
**Treatment cost/year,** euros	37·437€	40·432€	43·428€	18·717€	20·216€	21·714€
